# Clinical and Laboratory Outcomes of Angled Screw Channel Implant Prostheses: A Systematic Review

**DOI:** 10.1055/s-0041-1740298

**Published:** 2022-02-21

**Authors:** Vanya Rasaie, Jaafar Abduo, Mehran Falahchai

**Affiliations:** 1 Department of Prosthodontics, Dental Research Center, Dentistry Research Institute, Tehran University of Medical Sciences, Tehran, Iran; 2 Department of Prosthodontics, Melbourne Dental School, Melbourne University, Melbourne, Australia; 3 Department of Prosthodontics, Dental Sciences Research Center, School of Dentistry, Guilan University of Medical Sciences, Rasht, Iran

**Keywords:** angulated screw channel, screw-retained prosthesis, computer aided design- computer aided manufacturing, implant

## Abstract

The purpose of this systematic review was to evaluate the clinical and laboratory outcomes of angled screw channel (ASC) restorations and to summarize the influencing factors. An electronic search of the English language literature was performed in four databases and enriched by manual searches. Retrieved studies were screened against the predefined exclusion and inclusion criteria. Eight clinical and seven laboratory studies were eligible for the analysis. The risk of bias for included observational studies was performed using the Newcastle–Ottawa quality assessment scale. Laboratory studies quality assessment method was adapted from previous published systematic reviews. Two clinical studies focused on technical outcomes and the rest reported the biological outcomes of the ASC restorations. Out of the seven laboratory studies, two studies investigated the fracture resistance of ASC restorations, four studies evaluated the reverse torque value of the nonaxially tightened screws, and one study evaluated both variables. The present review revealed that while the performance of ASC restorations is promising in short-term clinical studies, the evidence of their long-term reliability is still lacking. The laboratory studies indicated comparable fracture resistance results of the ASC restorations with the straight screw channel restorations. In addition, factors, such as initial torque value, configuration of the screw driver, screw design, abutment system, and the angulation of screw channel, were shown to influence the screw resistance to loosening.

## Introduction

The implant-supported prosthesis is a successful strategy to restore dental esthetics and function. 1 The definitive restoration can be attached to the implant by cement-retention or screw-retention mechanisms. 2 While cementing implant restorations appears a simple procedure, clinical evidence suggests an association between biological complications and excess cement. 3 4 5 6 Moreover, the difficulties in their maintenance and their retrievability have made screw-retention mechanism a more preferred choice. 3 5 7 However, implants are not always placed in favorable positions for screw-retained restorations. This can be frequently encountered in the anterior maxilla due to centripetal resorption pattern and concave bone configuration. Further, angulated implant placement in the posterior regions can be indicated to avoid critical structures like maxillary sinus or mandibular canal. The treatment options to correct implant angulation may involve the use of an angled abutment with a cement-retained restoration, or surgical bone augmentation to allow for more ideal implant placement. 8 Alternatively, angle correction via intermediate abutments or restorations retained by a lateral screw could be considered. However, these systems have been shown to increase the treatment complexity, maintenance burden, and incur additional costs. 8 9

Almost two decades ago, angled screw channel (ASC) abutment design (Dynamic Abutment; Talladium International Implantology) was introduced to allow restoring angulated implants with simple direct to fixture screw-retained restoration. The ASC design uses a screw with a hexalobular head shape that can be engaged with a hexagonal faceted sphere screwdriver at various angles between 0 and 28 degrees with 360-degree freedom of rotation. 10 This allowed tightening the abutment screw at an orientation different from the center axis of the implant. Earlier, ASC abutments were casted on a hemisphere base via a burnout sleeve that could be rotated freely to direct the screw access channel away from the area of concern. 10 Additionally, this concept was limited to certain implant systems. Recently, advancements in implant software programs and manufacturing systems have made it possible to design and fabricate ASC restorations digitally. Moreover, prefabricated titanium bases incorporating the ASC are available from some implant manufacturers. The versatility of ASC has been confirmed by a cone bean computed tomography (CBCT) analysis study that showed screw-retained restorations are achievable with the use of ASC abutments in 76% of cases in the anterior maxilla. 11 Despite the increased popularity, the efficiency and survival of the ASC systems remain unclear. The purpose of the present systematic review was to investigate laboratory and clinical outcomes of ASC restorations through the available literature to determine their survival and the influencing factors.

## Methods

This systematic review adapted the Preferred Reporting Items for Systematic Reviews and Meta-Analysis (PRISMA) statement. The aim was to answer the following focused question “what are the outcomes of implants restored with angled screw channel prostheses?”

The main search strategy was developed for PubMed ( Table 1 ) and supplemented with additional search in Science Direct, Scopus, and Cochrane Library. The search was conducted in March 2021 and updated in May 2021. The search aimed to identify all the available clinical and laboratory studies on ASC. The inclusion criteria were peer-review publication, prospective or retrospective clinical study with at least 1-year observation period after implant restoration, clinical study that evaluated biological and/or mechanical outcomes, clinical study that clearly listed outcome related to ASC restorations, and laboratory study that evaluated ASC performance variables with clinical relevance. The studies were excluded if they were not in English language, the ASC-related data could not be determined, or the restorative stage details were not clearly stated. Duplicated articles from different searches were discarded by a reference manager software program (Endnote X9, Clarivate Analytics, Philadelphia, Pennsylvania, United States). Following this, the titles and abstracts were screened. The articles of interest were selected for full-text analysis and matching against the inclusion criteria. Further, the reference lists of the included studies were manually searched. Quality of the selected clinical studies was scored with Newcastle–Ottawa scale for nonrandomized studies ( Table 2 ) which were designed to assess the quality of cohort studies based on selections of exposed and nonexposed cohorts, comparability influenced by the controls of risk factors, and completeness of outcomes. 12 The risk of bias of the included laboratory studies were assessed by using an adaptation of the methods applied in two previous systematic reviews. 13 14 Descriptions of the following parameters were used to assess each article's risk of bias ( Table 3 ): sample size calculation, presence of a control group, type of component used (genuine/nongenuine), statistical analysis performed, reliable analytical methods or statistical indicators, blinding of the evaluation assessors, and utilizing clinically relevant restoration material. A “yes” was assigned where the parameter was reported in the text and a “no” if the information was absent. The risk of bias was classified according to the sum of “yes” received as follows: 1 to 3 = high, 4 to 5 = medium, and 6 to 7 = low risk of bias.

**Table 1:** Search strategy

Search strategy	Query
Population: screw-retained implant supported restorations	(((Screw-retained[All Fields] AND implant[All Fields])) OR (“prostheses and implants”[MeSH Terms] OR (“prostheses”[All Fields] AND “implants”[All Fields]) OR “prostheses and implants”[All Fields])) OR (screw-retained[All Fields] AND restoration[All Fields])
Intervention: ASC restorations	((((abutment screw) AND (off[All Fields] AND “axis”[All Fields]))) OR ((“single”[All Fields] AND implant[All Fields] AND restoration[All Fields]))) AND ((((((angled[All Fields] AND “screws”[All Fields] AND channel[All Fields])) OR (angulated[All Fields] AND screws”[All Fields])) OR (non-axial[All Fields] AND “screws”[All Fields] AND channel[All Fields])) OR (Abutment[All Fields] AND “screws”[All Fields] AND channel[All Fields])) OR (two[All Fields] AND piece[All Fields] AND abutment[All Fields]))
Outcome:	(((((reverse[All Fields] AND (“torque”[MeSH Terms] OR “torque”[All Fields]))) OR (technical[All Fields] AND (“complications”[Subheading] OR “complications”[All Fields]))) OR (mechanical[All Fields] AND complication[All Fields])) OR (fractures[All Fields] AND resistance[All Fields])) OR “survival rate”[MeSH Terms]

**Table 2:** Quality assessment of selected clinical studies using Newcastle–Ottawa scale for cohort studies

Quality assessment criteria	Acceptable	Greer et al 23	Anitua et al 25	Tallarico et al 27	Friberg and Ahmadzai 24	Pol et al 22	Anitua et al 26	Shi et al 28	Nastri et al 29
Representativeness of exposed cohort?	Representative of average adult in community	1			1				
Selection of nonexposed cohort	Drawn from same community as exposed cohort								
Ascertainment of exposure	Secured records	1	1	1	1	1	1	1	1
Demonstration that outcome of interest not present at start of the study	Yes	1	1	1	1	1	1	1	1
Study controls for the degree of screw access angulation	Yes		1				1	1	
Study controls for additional risk factor?	restorative material	1	1	1		1	1	1	
Assessment of outcome	Secure records	1	1	1	1	1	1	1	1
Follow-up long enough	Follow-up (>1 year)			1			1		1
Adequacy of follow-up	Small number of subject loss	1	1	1		1	1	1	1
Overall quality score (maximum = 9) >7: good/5–7: fair/< 5 poor		6	6	6	4	5	7	6	5

**Table 3:** Quality assessment and risk of bias of laboratory studies considering aspects (reported in “Materials and Methods” section)

Author (year)	Sample size calculation	Control group	Genuine component used	statistical analysis performed	reliable analytical methods or statistical indicators	blinding of the evaluation assessors	Utilization of clinically relevant restoration material	Risk of bias
Goldberg et al (2019) 21	No	Yes	No	Yes	Yes	No	No	High
Hu et al (2019) 20	Yes	Yes	Yes	Yes	Yes	No	NA ^•^	Medium
Opler et al (2019) 18	No	Yes	No	Yes	Yes	No	NA	High
Swamidass et al (2021) 17	No	Yes	Yes	Yes	Yes	No	Yes	Medium
Drew et al (2020) 16	No	Yes	Yes	Yes	Yes	No	Yes	Medium
Garcia-Hammaker et al (2021) 15	Yes	Yes	Yes	Yes	Yes	No	Yes	Low
Mulla et al (2021) 19	No	Yes	Yes	Yes	Yes	No	Yes	Medium

Abbreviation: NA, not applicable.

## Results

**Fig. 1:**
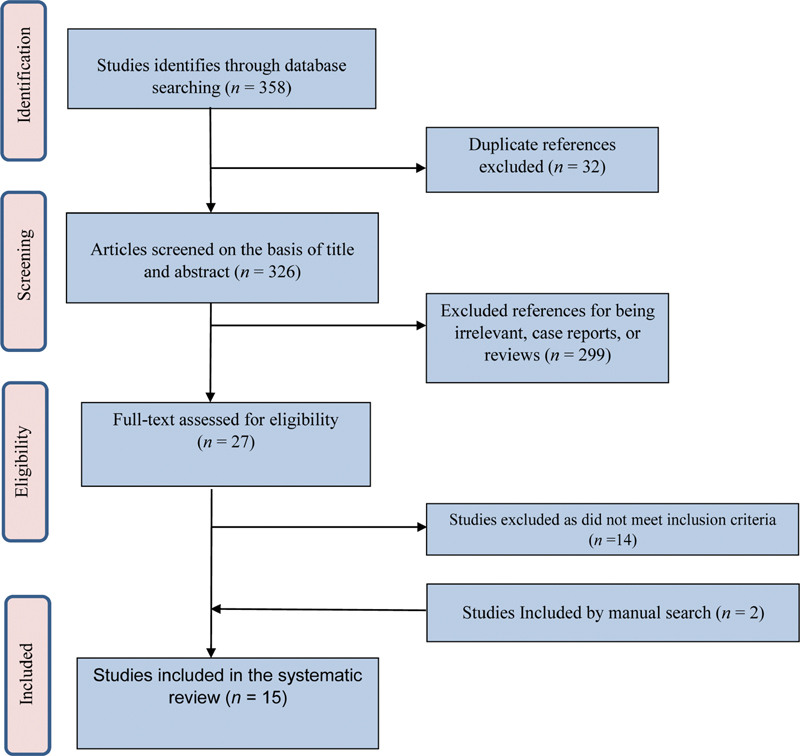
Flowchart for search process according to PRISMA guideline. PRISMA, preferred reporting items for systematic reviews and meta-analyses.

The initial electronic search yielded 358 publications. After elimination of duplicates, 326 remained for title and abstract review. Twenty-seven articles were selected for full-text analysis, 13 of which were laboratory and clinical studies that fulfilled the inclusion criteria. Fourteen studies were excluded as listed in Table 4 along with the reasons of exclusion. The supplementary manual search through the bibliography of the included studies yielded two additional articles. Therefore, 15 publications were eligible for the current review ( Fig. 1 ). The included studies were published between 2017 and 2021, with the majority being published in the past 2 years. Out of seven laboratory investigations, two studies evaluated the fracture strength of the zirconia crowns with ASC ( Table 5 ), 15 16 four studies evaluated the reverse torque value of nonaxially tightened screws ( Table 6 ), 17 18 19 20 and one examined both variables. 21 Only four studies reported the exact direction of force in their investigations that was 30 degrees for all of them. 15 17 19 21 A total of eight nonrandomized cohort studies (four prospective and four retrospective) reported the outcomes of 281 implants restored with ASC restorations in 254 patients ( Table 7 ). 22 23 24 25 26 27 28 Of these, two investigations focused on technical complications 23 25 and the rest reported the technical and biological outcomes of ASC restorations. 22 24 26 27 28 Six studies included only single-crown restorations 22 23 24 27 28 and two studies included partial or full arch restorations. 25 26 All but two studies reported cumulative survival rates after at least 1 year of loading.

**Table 4:** Reasons for exclusion of discarded studies after full-text analysis

Reasons for exclusion	Did not evaluated ASC restorations	Not indicative of variables with clinical relevance	No data about prosthetic stage of treatment
Excluded studies	Anitua et al 40 Chen and Pan 41 Guljé et al 42 Hotinski and Dudley 43 Lin et al 30 Menéndez-Collar et al 44 Mokhtarpour et al 45 Paolantoni et al 46 Vélez et al 47	Edmondson et al 11 González-Martín and Veltri 48 Farré-Berga et al 49 Farronato et al 50	Wang et al 51

Abbreviation: ASC, angled screw channel.

**Table 5:** Detailed data of included laboratory studies (fracture/fatigue studies)

Author (year)	Sample size	Implant system	Abutment system	Restoration material and design	Evaluated screw channel angulation (degree)	Tightening torque value	Aging (cyclic loads and thermocycles)	Fracture test	Results	Mode of failure
Goldberg et al (2019) 21	*n* = 7	External hexagon implants, Osseotite; Zimmer Biomet (Fixture)	1. Dynamic Abutment (DA); (Dynamic Abutment Solutions.) 2. UNISG Abutment with gold square screw (GS); (Zimmer Biomet.)	Full contour casted Ni–Cr crown Maxillary central incisor	GS: 0 DA: 0 DA: 20 DA: 28	GS: 0, 35 Ncm DA: 0,20, 28, 25 Ncm	Cyclic load: in dual axis mastication simulator under axial load of 40 N for 1200,000 cycles. (all specimens were retightened at 9,205 cycles)	Fracture strength was tested by a universal testing machine under compressive load at 30-degree angle until failure	Fracture strength (N): GS-0 degrees: 989.01 DA-0 degrees: 869.59 DA-20 degrees: 715.88 DA-28 degrees: 789.84	Screw fracture: GS-0 degrees: 2, DA-0 degrees:2, DA-20 degrees: 1, DA-28 degrees:0. All crowns remained intact but implant platform was severely deformed or fractured
Drew et al (2020) 16	*n* = 5	4.3 mm × 10 mm, NobelActive, Nobel Biocare (Fixure)	1. Nobel Procera, (Nobel Biocare) with a titanium adapter.	CAD/CAM monolithic zirconia crown Maxillary central incisor	0 25	35 Ncm		Off-axis compressive sinusoidal fatigue load cycles of 10 to 200 N at 15 Hz with a maximum number of 334,800 (250,000 cycles is equivalent to 1 year of clinical service)	Mean number of cyclic loads for incisal-cervical fractures/catastrophic failure: ASC abutments: 16,650/83,385 SSC abutments: 135,270/212,940	All ceramic fracture occurred in the cingulum area in an incisal-cervical direction. The crack occurred from the apical part of the screw access opening to the level of titanium adapter
Garcia-Hammaker et al (2020) 15	*n* = 10	Conical connection regular platform, Nobel Biocare (Analog)	Nobel Procera, (Nobel Biocare) with a titanium adapter	CAD/CAM monolithic zirconia crown Maxillary central incisor	0 25	35 Ncm	Not conducted	Perpendicular compressive static force on samples mounted with 30-degree angulation (2-mm below the incisal edge) until failure, using a universal testing machine	Mean value of the load to failure: 0 degrees: 534.05 25 degrees: 215.49 Maximum load: 0 degrees: 762.70 25 degrees: 420.51	25-degree zirconia abutments: fracture at the most apical portion of the zirconia piece with some minor damage to the screw head. Eight specimen showed loss of screw channel continuity

**Table 6:** Detailed data of included laboratory studies (reverse torque studies)

Author (year)	Studied samples and sample size	Implant system	Abutment system	Restoration material and design	Evaluated screw channel angulation (degree)	Tightening torque value	Aging (cyclic loads and thermocycles)	Results
Goldberg et al (2019) 21	*n* = 7	External hexagon implants, Osseotite; Zimmer Biomet (Fixure)	1.Dynamic Abutment (DA); (Dynamic Abutment Solutions) 2. UNISG Abutment with gold square screw (GS); (Zimmer Biomet.)	Full contour casted Ni–Cr crown Maxillary central incisor	GS-0 DA-0 DA-20 DA-28	GS-0: 35 Ncm DA-0,20,28 degrees: 25 Ncm	Cyclic load: in dual axis mastication simulator under axial load of 40 N for 1,200,000 cycles (all specimens were retightened at 9,205 cycles)	Mean difference between baseline and removal torque (ΔRT) values: GS-0 degrees: −1.04 DA-0 degrees: 1.09 DA-20 degrees: −0.51 DA-28 degrees: −2.57
Hu et al (2019) 20	*n* = 16	regular platform; iMilling, Chantilly, VA (Analog)	4.3 mm regular platform S-Link abutment; iMilling, Chantilly, VA	No restoration fabricated	0 10 20	35 Ncm	Not conducted	Mean reverse torque values: 0 degrees: 31.16 10 degrees: 32.07 20 degrees: 30.09
Opler et al (2019) 18	*n* = 10	NobelActive, Regular platform; Noble Biocare	Dynamic Abutment, Talladium International Implantology	No restoration fabricated	0 10 15 25 28	25 Ncm	Not conducted	Mean input torque/ mean reverse torque value (Ncm) 0 degrees: 25/22.18 28 degrees: 25/17.44 Mean input torque/mean output torque (the ultimate delivered torque) value range for all study groups (Ncm): 24.97 to 25.01/24.36 to 25.81
Swamidass et al (2020) 17	*n* = 10	Nobel Biocare Replace Conical Connectio 4.3 mm ×11.5 mm; Nobel Biocare (Fixure)	1.Nobel Biocare Gold-Adapt (GA) 2. Nobel Biocare SSC (NB) 3. Nobel Biocare ASC (NB) 4.Dynamic Abutment Solution (DA) 5.Core3dcentre angle correction (C3D)	CAD/CAM monolithic zirconia crown Maxillary central incisor	NB-0 GA-0 NB-20 DA-20 C3D-20	NB-20 degrees, NB-0 degrees, GA-0 degrees: 35 Ncm DA-20 degrees: 25 Ncm C3D-20 degrees: 20 Ncm	Thermocycle: 5°C to 55°C for 5,000 cycles. Cyclic load: 0 N to 100 N, 30 degrees to the implant long axis at 10 Hz for 1 million cycles (equivalent to 1 year of masticatory cycle)	Median of torque removal values: precyclic loading/postcyclic loading, median percentage torque loss (Ncm) NB-0 degrees: −22.9/ – 20.6, 10.9% GA-0 degrees: −17.7/− 11.0, 35.9% NB-20 degrees: −23.9/− 19.4, 20.8% DA-20 degrees: −14.1/− 8.95, 31.9% C3D-20 degrees: −12.8/− 7.75, 34.5%
Mulla et al (2021) 19	*n* = 7	Nobel Replace Conical Connection 4.3 mm ×10 mm; Nobel Biocare (Analog)	1.Dynamic Abutment Solutions (DA) 2. AngleBase; Dess Dental Smart Solutions (DE) 3. Angled Screw Channel Solutions; Nobel Biocare (ASC) 4. Universal Base; Nobel Biocare (UB)	CAD/CAM monolithic zirconia crown Maxillary central incisor	UB-0 DA-25 DE-25 ASC-25	DA: 25 Ncm DE, ASC, UB: 35 Ncm	Cyclic load: in masticatory simulation machine at 30° under a 200 N load at 2 Hz for 5 million cycles, (equivalent to 5 years of functional loading) The mastication simulator was stopped every 500,000 cycles to retighten any loose crowns, with each stop simulating a 6-month recall appointment (no specimen found loose. Thus no retightening was performed)	Mean of input torque deviation value (ITV) from the target value (ITV1/ITV2; Ncm): UB: 0/− 0.2 DA: −0.7/− 0.2 DE: −3.9/− 2 ASC: −4.1/− 2.1 Mean of RTV after 24 hours/after cyclic loading (Ncm): UB: 27/16 DA: 17/11 DE: 27/15 ASC: 26/18 Preload efficiency: UB: 48.5% DA: 43.8% DE: 46.8% ASC: 54.2%

**Table 7:** Detailed data of included clinical studies

Author (year)	Study type	Observation period	Number of patients	Number of restorations (unit)	Restoration material	Location in the arch	Implant system	Time and technique of implant placement	Angulation of screw access channel	Results
Greer et al (2017) 23	Retrospective	Mean: 216.3 days (range: 14–784 days)	60	84 Single crown	Nobel Biocare Procera ASC 90% Procera full contour zirconia 10%	Maxilla 90%/mandible 10% Incisor (>70%)/premolar (>20%)/canine (>5%)/molar (<5%)	Nobel active internal connection (64%) Nobel replace conical connection (36%)	Not reported	Not reported	96% with no complications. 4% with mechanical complications reported (a loose screw, a ceramic fracture, an implant failure)
Anitua et al (2018) 25	Retrospective split mouth	1 year	52	0 degrees: 55 Median number of units: 3 (2–12) ASC: 55 Median number of units: 3 (2–13)	CAD/CAM Cr–Co framework veneered with porcelain	0 degrees: maxilla: 21/mandible: 34 ASC: maxilla: 26/mandible:29 Premolar and molar regions	Dental implants, BTI Biotechnology Institute	Not reported	0 degrees ( *n* = 55) 15 degrees ( *n* = 46) 30 degrees ( *n* = 9)	Seven complication in experiment group (five porcelain fracture, one screw loosening, and one screw fracture) Four complications in control group (three porcelain fracture and one screw fracture)
Tallarico et al (2018) 27	Prospective case series	Up to 3 year (mean duration: 38.2 months)	10	23 Single crown	CAD/CAM zirconia framework veneered with feldspathic porcelain	Posterior maxilla	NobelReplace Conical Connection; Nobel Biocare AG	Delayed placement. flapless procedure and immediately loaded	Not reported	Prosthesis and implant cumulative survival rate: 100% Mean marginal bone level: Placement and loading: 0.29 ± 0.34 mm/1-year follow-up: 0.37 ± 0.32 mm/2-year follow-up: 0.38 ± 0.33 mm/3-year follow-up: 0.50 ± 0.42 mm
Friberg and Ahmadzai (2019) 24	Prospective	Up to 1 year	47	51 (ASC: 42)/22 implants were followed Single crown	Not reported	Maxillary incisors and canine	NobelParallel CC, Nobel Biocare Narrow platform: 27 Regular platform:24	4 immediate placement and 47 late placement, all with two-stage procedure. 14 implant sites were bone augmented	Not reported	Implant cumulative survival rate: 98.0% Mean marginal bone loss: 0.41 ± 0.36 mm No complication reported in relation to the ASC restorations.
Pol et al (2020) 22	Prospective case series	1 year	30	30 Single crown	CAD/CAM full contour zirconia crowns with metal adapter	Maxilla: 12 Mandible: 18 Molars	NobelParallel CC, Nobel Biocare Regular platform	Delayed placement. Two-stage procedure	Not reported	Prosthesis success rate: 100% Implant survival rate: 100% Mean marginal bone loss: 0.16 ± 0.26
Anitua et al (2020) 26	Retrospective controlled split mouth	Mean duration: 45.5 ± 15.02 months	22	0 degrees: 34 Partial: 88.2% Complete: 11.8% ASC: 34 Partial: 94.1% Complete: 5.9%	CAD/CAM Cr–Co framework layered with porcelain	0 degrees: maxilla: 55.9%/mandible: 44.1% ASC: maxilla: 47.1%/mandible: 52.9% Anterior and posterior regions of the arch	Dental implants, BTI Biotechnology Institute	Delayed placement. Surgical technique: not reported	0 up to 30 degrees	Survival rate: 100% Mean marginal bone loss: −0.29 ± 0.51 mm
Shi et al (2020) 28	Prospective	1 year	44	CR: 20 ASC: 24 Single crown	CR: zirconia base restoration veneered with ceramic on a custom-made zirconia abutment. ASC: NobelProcera zirconia framework veneered with ceramic	Anterior maxilla	NobelActive internal connection and NobelReplace conical connection, Nobel Biocare	Immediate flapless placement and delayed loading	Mean angulation of screw channel: 13.7 degrees (0–25 degrees)	Implant survival rate: 100% Mean marginal bone loss: ASC: 0.31 ± 0.30 mm CR: 0.41 ± 0.38 mm Mechanical complication rate: ASC: 13.0% (2 screw loosening, 1 ceramic fracture (fracture between zirconia coping and veneer material) CR: 5.0% (1 loss of retention)
Nastri et al (2021) 29	Retrospective	Minimum 2 years ASC: 36.4 ± 10.3 months CR: 52.3 ± 5.7 months	20	CR with custom abutment: 10 ASC: 10 Single crown	Not reported	Central incisor: 4 Maxillary lateral incisor: 8 Maxillary canine: 4 Maxillary first premolar: 1 Mandibular lateral incisor: 3	Nobel Biocare	Not reported	Not reported	No mechanical complication reported Total white esthetic score/pink esthetic score: baseline: ASC: 16.6 CR: 17.3; follow-up: ASC: 16.2; CR: 17.1 Mean MBL: ASC: −0.22 ± 0.19 mm/CR: −0.29 ± 0.11 mm Mean bleeding on probing: Baseline: ASC: 0/10 CR:2/10 Follow-up: ASC: 2/10 CR: 2/10 Mean probing depth: baseline: ASC: 3.7 mm CR: 3.3; follow-up: ASC: 4 CR: 3.7

Abbreviation: ASC, angled screw channel; CC, conical connection; CR, cement retained.

Drew et al 16 and Garcia-Hammaker et al 15 examined the fracture strength of the two piece CAD/CAM monolithic zirconia crown with 25-degree ACS by using cyclic and static loads, respectively. Drew et al 16 showed no statistically significant difference in the mean number of cyclic loads to failure between ASC and straight screw channel (SSC) crowns. However, the SSC crowns survived a greater number of cycles prior to failure. Garcia-Hammaker et al 15 reported a significantly higher mean fracture load by 2.4 times and maximum load before failure by 1.8 times for the SSC crowns. Despite the superior fatigue loading of SSC, the specimens in both groups resisted the physiologic loads and failure occurred at loads that resembled a parafunctional situation. Goldberg et al 21 used external connection hexagon implants for the comparison of the fracture strength of SSC gold screw abutments with 0, 20, and 28 degrees of Dynamic abutments casted to nickel–chromium (Ni–Cr) crowns, after aging in a mastication simulator. The study reported screw fracture before implants' mechanical failure in 17.8% of specimens with only 0.03% of fractures attributed to the ASC restorations. Furthermore, no significant difference was reported among the fracture strength values of the studied groups.

In the same study, Goldberg et al 21 compared the reverse torque value (RTV) of the nonaxially tightened dynamic abutment screws (DAS) with 0-degree DAS and SSC gold screw. Although not statistically significant, the 0-degree DAS demonstrated the highest RTVs, while the 28-degree DAS showed the lowest values. The study suggested that increased off-axis loading resulted in higher tensile forces on abutment screws.

Hu et al 20 showed that the screwdriver insertion angulation has a significant impact on the RTVs of the abutment screws tightened in 0-, 10-, and 20-degree angles. The lowest mean RTV was in the 20-degree group and was described as the loss of applied torque due to increased screwdriver angle from the action of force. The highest mean RTV was shown in 10-degree specimens. This was explained by the possibility of a more intimate engagement of the screwdriver tip and the abutment screw at 10-degree angulation for the chosen system. Another study investigated the effect of screwdriver insertion angulation on the RTVs, input torque values delivered at various angulations (0, 10, 15, 25, and 28 degrees), and the transmitted output torque values in five different angulations. While the study revealed no significant difference in input torque values among the groups, the mean RTV between the 0- and 28-degree groups was significantly different with lower values for the 28-degree group. Regarding the mean output torque values, which was measured by the strain induced in the screw body, the results revealed no significant difference between 0- and 15-degree angulations. However, significantly lower output torque transmission to abutment screws was found when the angulation increased to 25 and 28 degrees. Furthermore, photography of the specimens revealed evidence of wear at the screw head predominantly with the 28-degree group, suggesting slipping of the driver. 18

Swamidass et al 17 investigated the differences in RTVs of ASC abutments from different manufacturers before and after simulated aging by temperature and cyclic loading. The RTV was measured 10 minutes after tightening as the initial value and subsequent to aging as the ultimate value. The percentage of the differences of RTVs was calculated and analyzed. In general, while the systems with higher initial torque value showed lower percentage torque loss, the differences between the 0- and 20-degree groups were not significant. On the other hand, when the abutment screws and implant were from the same manufacturer, no significant difference between the SSC and ASC groups was identified. In contrast, differences between the SSC group with genuine abutment screws and the ASC group with abutment screws from alternate brands were significant. An additional finding was the wear of the screw head against the zirconia crown in groups with friction fitted two-piece zirconia abutments (Nobel Biocare). Congruent with this study, Mulla et al 19 evaluated a higher magnitude of cyclic forces for a longer duration of time on 25-degree access channel restorations from three different manufacturers. RTVs were measured 24 hours after initial tightening and after simulated 5 years of functional load. Additionally, their study analyzed the deviation of the input torque value from the target value recommended by the manufacturer. Two hexalobular systems (Nobel Biocare and Dess Dental Smart Solutions) delivered significantly lower input torque values at 25 degrees compared with the SSC group. Nonetheless, their measured RTVs were not significantly different from the RTVs of SSC group. The 25-degree dynamic Ti-base system revealed insignificant input value torque deviation compared with 0-degree group. Unlike the other three study groups, this hexalobular system exhibited a high amount of torque loss at both times of RTV measurement. The statistical analysis of this study revealed significant differences in mean RTVs for both 24-hour RTV and after cyclic loading among all groups. In addition, the RTV means before and after cyclic loading for each group were significantly different. Furthermore, out of five catastrophic failures reported with the 25-degree groups, three were related to zirconia fracture initiated from the area surrounding Ti-base. Other two failures were Ti-base and screw head fracture in systems with cemented two-piece zirconia abutment. Overall, no significant difference was found in the survival rate among the groups.

Overall outcomes from the seven clinical studies showed a high survival rate for both the ASC restorations (88–100%) and the dental implants (98–100%) with low mean marginal bone loss (MBL; 0.16–0.41 mm). However, the majority of the studies were conducted for a short duration of time (1 year). Ceramic fracture was the most frequent complication that was reported in three studies (seven events), 23 25 28 mostly related to 15-degree metal–ceramic multiunit restorations with equal incidents in both arches (two in mandible and two in maxilla). Poor occlusal management was the attributed causes of ceramic fracture in the Greer et al study. 23 Screw loosening was reported in three studies (four events), 23 25 28 and screw fracture was reported in one study (one event). 25

Two studies included data for ASC and SSC restorations. 25 26 These studies evaluated multiunit metal ceramic fixed prosthesis in the posterior region (premolar and molar) of both arches, whereas the abutment screws were tightened either in axial or nonaxial direction. During follow-up, a total of 11 technical events were reported, 7 in the ASC group and 4 in SSC group ( Table 6 ). However, analysis of the results indicated that neither the frequency of technical complications nor the implants' survival and MBL were affected by the angulation of the screw channel. Tallarico et al 27 used ASC zirconia abutments for immediate restoration of tilted implants placed in the posterior region of the atrophic maxilla. Although the studied sample size was limited, no biological or technical complications were reported in the 3-year follow-up. The authors suggested that the combination of tilted implants and ASC abutments might be a safe alternative to maxillary sinus floor augmentation procedures when patients refuse additional surgical procedures. Friberg and Ahmadzai 24 and Pol et al 22 evaluated the ASC restorations with conical connection implants for replacement of single missing teeth in incisor/canine and molar region, respectively. None of the studies indicated any complication after 1 year of function. Shi et al 28 compared the technical and biological outcomes of ASC and cement-retained single restoration in anterior maxilla after 1 year of loading. Their report indicated that while the difference in MBL was insignificant among the two study groups, bleeding on probing percentage was significantly higher in the cement-retained group. In addition, four events of technical complications were reported. Screw loosening and ceramic chipping were the complications associated with the ASC cohorts. Contrary to these findings, Nastri et al 29 reported no mechanical complication and no significant difference in bleeding on probing among their study cohorts (cement-retained versus ASC single crowns) through the 2-year follow-up. Additionally, the differences in probing depth, mean MBL, and white esthetic score between the two study groups were insignificant. However, the cement-retained restorations had significantly higher pink esthetic scores both in baseline and follow-up recordings.

## Discussion

This systematic review critically appraised the existing evidence from the laboratory and the clinical studies on the implant-supported ASC restorations. Concerning laboratory studies, fracture strength and screw resistance to loosening were investigated. According to fracture strength studies, the ASC restorations appear to fail at less cycles of loads and lower forces than SSC. However, since they sustained the expected physiological forces, the ASC restorations were considered mechanically comparable with SSC counterparts. 15 16 21 Fractographic examinations of anterior two-piece zirconia abutments revealed that critical cracks were initiated in the cingulum from the most apical part of the screw access channel in the friction-fitted system. 15 16 19 Consistent with this finding, some clinical studies have reported early catastrophic failures with the same pattern in two-piece friction fitted zirconia abutments in anterior and premolar SSC restorations. 30 31 As the screw head seats on the internal surface of the zirconia restoration, hoop stresses and/or incompatible hardness between the zirconia and titanium components may be responsible for such pattern of failure. 16 17 19 This could suggest that the abutment-titanium base interface design might play more considerable role than the ASC in the long-term performance of the hexalobular systems. On the other hand, as the angulation of the screw channel is increased, the bulk of the palatal walls of anterior zirconia abutments is reduced. 15 16 This could lead to a weak point in zirconia restorations where the thickness my reach to less than 0.7 mm. 32 Thus, precaution should be taken when using ASC until more robust evidence is available on the interaction between the angulation amount and abutment thickness. Since ASC restorations were mostly required in the anterior maxillary region, 11 the investigated restoration materials by laboratory studies (monolithic zirconia or full-contour Ni–Cr crowns) may not be fully representative of the clinical application of ASC in highly demanding esthetic clinical situation. Therefore, further studies are required to determine the reliability of other restorative materials with ASC.

When resistance to screw loosening is considered, studies showed that the RTV was influenced by initial torque value, 17 19 configuration of the screwdriver, 20 screw design, 17 19 abutment system, 17 19 and angulation of the screw channel. 17 18 19 20 21 As the torque is applied to an abutment screw, it elongates and the threaded surface elastically deforms. Adequate screw elastic elongation, referred to as preload, could secure the implant-abutment joint by appropriate clamping forces. 33 A reduction in the input torque could compromise the screw joint by reducing the applied preload. 34 A consistent outcome of the two studies investigated various ASC systems indicated that the higher the initial torque value, the less susceptible the screw to loosening. 17 19 Although the measurement methods were different, two studies showed that the actual torque delivered to the screw is reduced when the insertion angle of the screwdriver exceeds 15 degrees (25 and 28 degrees). 18 19 However, RTVs of screws from different systems showed variable behaviors. For example, while 25-degree Nobel Biocare and 25-degree Dess abutment screws performed similarly, 19 the 28-degree DAS exhibited a significant 23% reduction in RTV when compared with the 0-degree control group. 18

Intimate engagement of the screwdriver and screw head showed to play a role in the amount of the delivered torque. This is further influenced by the screwdriver's sphere and facet design, as well as, the surface treatment of the screw head. 17 18 19 20 Likewise, difficulty in the engagement of the screwdriver, which was reported with certain systems, led to stripping and wear of the screw head that might impact the crown retrievability and the usability of the screws after multiple tightening in long duration of time. 17 18

The use of nongenuine component can influence the RTV and may jeopardize the joint stability of the implant-abutment complex. 17 A proper match and integration of the components within an implant system is important, especially when using implants with conical/internal connection as shown by the included studies. 35 Further investigations is required to evaluate the RTV with other types of implant connection.

The majority of the included clinical studies reported no major complications after 1 year of treatment of posterior regions with conical connection Nobel Biocare implants and ASC restorations. 22 25 26 27 Consistent with these, the anterior ASC restorations revealed favorable clinical performance as well. 23 24 29 Technical complications were reported in three studies 23 25 28 with no significant differences between ASC and SSC or cement-retained restorations. 25 26 28 A previous clinical study with up to 9 years of follow-up has reported that two-piece zirconia SSC abutments with bonded titanium insert can be a suitable option for anterior and premolar region. However, in the molar area, the use of the same abutment without a complete metal-to-metal connection platform (friction-fitted titanium insert) to support the restorations have led to a high incidence of fracture. 30

The data on MBL around the ASC restorations revealed favorable outcomes. Previous systematic reviews have indicated that changes in the crestal bone level is not significantly affected by angulated implant placement as compared with axial placement. 36 37 However, other factors related to the abutment design may affect the amount of MBL, such as the abutment height, and contour, 38 and repeated disconnection and reconnection of the abutment. 39 In the present systematic review, this complementary information was mostly missing. In addition, although the MBL in ASC and SSC were comparable, the number of studies comparing them was scares.

While ASC restorations show promising short-term results, some clinical questions are yet to be answered. For example, there is a need to determine their long-term clinical performance, cost-effectiveness, and the management of their complications. Thus, the availability of ASC should not be a justification for injudicious angular implant placement.

## Limitations

The present systematic review is limited with the fact that few clinical studies provided clear comparison between ASC and SSC. In addition, the fair quality of the majority of clinical studies and high-to-medium risk of bias of the laboratory mandate the need for stronger future evidences. Another limitation is that the clinical data were incomplete regarding the amount of angle correction. Moreover, most of the clinical studies had only 1-year duration of observation which does not reflect the long-term performance of ASC. Thus, the current evidence for use of ASC is limited and a more robust clinical guidance on the application of ASC abutments is needed.

## Conclusion

Within the limitation of this systematic review, the following inferences can be drawn:

1. According to laboratory studies, the fracture resistance of SSC and ASC restorations were comparable. The incidence of screw loosening might be lower in ASC systems with higher insertion torque and genuine components.
2. According to clinical studies, although the ASC restorations demonstrated favorable performance in anterior and posterior regions of the mouth in short-term, evidence is insufficient to determine their long-term survival.
